# The Virtual International Day of the Midwife: A model for digital knowledge translation

**DOI:** 10.18332/ejm/136048

**Published:** 2021-05-03

**Authors:** Cecilia M. Jevitt, Jane F. Houston, Alison Anderson, Susana Ku Carbonell, Halima M. Abdul

**Affiliations:** 1 Midwifery Program, Department of Family Practice, Faculty of Medicine, The University of British Columbia, Vancouver, Canada; 2 Midwifery and Women’s Health, Frontier Nursing University, Clearfield, United States; 3 St. James University Hospital, Leeds, United Kingdom; 4 Faculty of Health Sciences, McMaster University, Hamilton, Canada; 5 Midwifery Department, Cardiff University, Cardiff, United Kingdom; 6 Department of Nursing Science, Ahmadu Bella University, Zaria, Nigeria

**Keywords:** midwifery, birth, virtual conference, knowledge translation, digital media, carbon footprint

The COVID-19 pandemic abruptly halted knowledge translation of new research findings through in-person meetings and continued to disable national and international person-to-person conference networking for a two-year period. This braking effect propelled the use of existing communication technologies including social media platforms and video conferencing for knowledge translation. The visionary Conference Committee of the Virtual International Day of the Midwife (VIDM) began developing online knowledge translation in 2009^1,2^.

The first VIDM meeting celebrated the May 5^th^ International Day of the Midwife by presenting a synchronous, open-access, online conference over 24 hours. Sessions averaged six participants in 2009 and grew annually until the 2020 pandemic year when World Health Organization presentations drew more than 250 participants per session. The 38 online presentations and 24 posters in 2020, in English and Spanish, exemplified the VIDM goals of giving researchers a forum to disseminate their work and providing an opportunity for midwives across the globe to engage with the latest evidence-based research. Participants came from 32 countries as diverse as Afghanistan, Bolivia, Iran, and Nigeria. The growth of the VIDM as a virtual conference pre-dated the rapid migration to online conferences forced by pandemic social isolation in 2020.

A volunteer committee of senior educators, technologists, midwives and student midwives from nine countries organize the VIDM conference through virtual meetings, email, online document sharing and social media. Co-founders Sara Stewart and Deborah Davis initially used Collaborate software from Otago University with Sarah broadcasting from her kitchen in New Zealand. Later University College Lillebaelt in Denmark donated teleconferencing software use. Frontier Nursing University currently supports use of its online teaching platform for the conference. Initial VIDM goals included increasing digital literacy among practicing midwives and enabling midwives in diverse geographical locations, many with inadequate access to education resources, to learn about international knowledge development around birth.

The VIDM Committee is passionate about strengthening professional midwifery and sharing midwifery knowledge, research and clinical practice to reduce disparities in educational opportunities, thereby improving maternal and newborn health outcomes across the world. The organizing challenges include covering world time zones over 24 hours, peer-reviewing multi-lingual abstracts, recording podcasts, and orienting all presenters and facilitators to be confident in giving virtual presentations. The VIDM supports novice speakers and students in presenting their work. The conference organizes around an annual theme, recruiting midwives and birth researchers whose work explores that theme, thus spreading emerging social values beyond countries of origin. ‘Birth Equity for All’ unifies the 2021 VIDM presentations^3^.

The social isolation imposed to reduce COVID-19 transmission had unanticipated global benefits. The ‘anthropause’ allowed freer animal movement and increased reproduction around the world^4^. Global air quality improved significantly following reduced air, automobile and public transportation use for work commuting and travel, the leading contributors to the carbon footprint of travel^5,6^. Travel bans prevented national and international in-person scientific meetings and further reduced the carbon footprint of science by reducing travel, paper use and conference promotionals^6-8^.

The VIDM is a model of environment-sparing international collaboration between midwives, student midwives, researchers and educators within a socially responsible non-commercial context^1^. The VIDM bridges the knowledge translation gaps caused by resource inequities and the pandemic isolation. The Conference Committee hopes that EJM readers will join the meeting this year at VIDM.org and then submit a proposal to be a presenter in 2022.
